# Aerodynamic limits of gliding flight in grey-headed albatrosses under variable wind conditions

**DOI:** 10.1242/bio.062556

**Published:** 2026-09-10

**Authors:** Janine Schoombie, Kenneth J. Craig, Lelanie Smith

**Affiliations:** Department of Mechanical and Aeronautical Engineering, University of Pretoria, Pretoria, South Africa 0021

**Keywords:** Grey-headed albatross, Soaring flight, Albatross aerodynamics, Wind-seabird interactions, Albatross flight performance, Computational fluid dynamics

## Abstract

Adult grey-headed albatrosses breeding on Marion Island, South Africa, experience highly variable near-ground wind vectors that can result in crash landings, some of which are fatal. This study quantifies the combinations of airspeed and wind direction that can lead to loss of lift or the generation of downforce sufficient to cause such crashes. Using previously developed three-dimensional grey-headed albatross body geometry, we conducted numerical simulations of this rigid geometry across a wide range of flight conditions defined by airspeed, angle of attack, and sideslip angle. Lift and aerodynamic efficiency (lift-to-drag ratio) were then evaluated to identify the conditions under which insufficient lift was produced. Simulations show that for airspeeds below 10 m s^−1^, the generated lift was lower than the average weight of an adult grey-headed albatross, with peak aerodynamic efficiency occurring at an angle of attack of approximately 5°. While the geometry generates lift effectively under either strong crosswinds or downdrafts alone, their combination can produce substantial downforce. Given that albatrosses preferentially exploit crosswinds at the meso-scale, transient gusts combining crosswind and downdraft components may force birds into the ground, particularly during low-altitude nest departure, increasing the likelihood of fatal crash landings.

## Teaser

**Summary:** By simulating airflow over a real albatross geometry, this study quantifies the crosswind and downdraft limits beyond which soaring albatrosses can no longer sustain flight.

## INTRODUCTION

Albatrosses are widely regarded as among the most efficient flyers in nature (see e.g. Catry et al., 2004); however, recent studies have demonstrated that they may be vulnerable under certain flight conditions. In particular, observations from a colony of grey-headed albatrosses (*Thalassarche chrysostoma*) on Marion Island (46°54′ S, 37°43′ E) indicate mortality events associated with adverse wind conditions (ca. 11% increase in expected annual adult mortality; Schoombie et al., 2023a,b). The Schoombie et al. (2023a,b) studies focused on an inland colony on Marion Island, comprising approximately half of the island's grey-headed albatross population. During the prevailing westerly winds (le Roux, 2008; Schoombie et al., 2025a), grey-headed albatrosses are exposed to highly variable flow conditions in both speed and relative inflow angle, which may reduce lift or induce downforce. However, wind measurements suggested that strong downdrafts may not be a dominant factor. This raises a key question: under which instantaneous combinations of wind speed and direction (vertical and lateral components) do a gliding grey-headed albatross fail to generate sufficient lift to sustain flight?

Other notable studies have shown that wind strongly influences albatross movements during oceanic flights (Weimerskirch et al., 2012, 2015), yet available aerodynamic data for grey-headed albatrosses are insufficient to assess which wind vectors could cause fatal terrestrial crash-landings. Variable winds can rapidly change aerodynamic loads, so quantifying how lift and drag respond to changing wind speed, angle of attack, and sideslip, in controlled experiments (Lentink and de Kat, 2014) or simulations (Omar et al, 2020), would improve understanding of fine-scale flight performance. Such data are typically collected when assessing aircraft performance capabilities (see e.g. Anderson, 2008) but are very rare for seabirds (with exceptions being Crandell and Tobalske, 2011, and (Lentink et al., 2007).

Typical aerodynamic data for gliding in natural systems are often derived using theoretical assumptions for drag to estimate aerodynamic efficiency and commonly assume that lift equals the mean body weight of the relevant species (Harvey and Inman, 2021). While these assumptions are reasonable for investigating flight performance under equilibrium gliding conditions, they provide limited insight into aerodynamic behaviour under adverse conditions induced by complex wind vectors. Detailed aerodynamic studies, as commonly performed in aircraft design, rely on a defined geometry for both experimental and numerical analysis. In a 2025 study, digital geometry was generated for a grey-headed albatross (Schoombie et al., 2025b) – the body, bill and a simplified tail were reproduced from several photographs of grey-headed albatrosses in flight and the wings were reconstructed by 3D scanning dried grey-headed albatross wings while replicating air flowing over the wing in a wind tunnel. Therefore, at the time of writing, this is the most accurate model of any albatross in steady gliding flight. The availability of this model makes it possible to conduct a computational fluid dynamics (CFD) study to investigate the aerodynamic forces on the body in many different flow conditions.

Despite being living birds rather than engineered systems, albatrosses are, compared to most other genera, especially well suited for fixed-wing aerodynamic studies. Albatross's wings experience high bending moments during flight, making flapping costly (Sakamoto et al., 2013), and they instead exploit wind to travel hundreds of kilometres with minimal flapping (Weimerskirch et al., 2000; Richardson et al, 2018). When albatrosses do flap, it is infrequent, ca. 2 flaps s^−1^ in black-browed albatrosses versus ca. 7 flaps s^−1^ in shearwaters (Sato et al., 2009; Sakamoto et al., 2013). Also, it is suggested that a ‘wing lock’ tendon keeps the wings fully extended without muscle use (Pennycuick, 1982).

In the current work, we look predominantly at the lift generation and aerodynamic efficiency of the rigid geometry of a grey-headed albatross in a range of airspeeds (U), angles of attack (α; angle between the bird's trajectory and the freestream flow measure in the vertical plane), and sideslip angles (β; angle between the bird's trajectory and the freestream flow measure in the horizontal plane). The aim is to identify the conditions under which the model generates sufficient lift to equal or exceed the average weight of an adult grey-headed albatross (ca. 3.3 kg; Schoombie et al., 2025b), and to determine the conditions that maximise aerodynamic efficiency, as a measure of maximum glide range. While other forces and moments were used to validate the CFD methods, it was not the focus of the present study, and no stability analyses were done here, rather, the CFD is validated against a test case at an appropriate Reynolds number. The study mainly answers the research question: At what U, α and β does the grey-headed albatross model (bill, body, wings and tail) generate sufficient lift for steady gliding?

Although the CFD model has some uncertainties and may not perfectly represent a real grey-headed albatross in flight, it will offer valuable insights into how varying wind speed and direction affect the birds' aerodynamics. The CFD results will be used to qualitatively assess when grey-headed albatrosses need to actively adjust their flight, such as changing direction or flapping, to maintain lift. Additionally, the results will provide a quantitative basis to understand how wind speed, crosswinds, and downdrafts reduce lift to the point of causing crash-landings.

A full description of five different grey-headed albatross geometry configurations is presented in Schoombie et al. (2025b): a wing alone (W), body-alone (B), body-tail (BT), bill-body-tail (BBT) and bill-body-wing-tail (BBWT) configuration. The configurations with separate body components (W, B, BT and BBT) were simulated initially for the purpose of investigating mesh dependency as well as inferring the contribution of each component to lift and drag production. The geometry configurations were treated as a fixed-wing aircraft. This fixed-wing assumption reflects the presumed optimal form of flight for a grey-headed albatross (i.e. minimise flapping and thus energy exertion). While albatrosses are well known for dynamic soaring, steady gliding flight aerodynamics still offer valuable insight into their soaring capability (Harvey and Inman, 2021).

This paper first validates the numerical methodology using a benchmark CFD case. The validated approach is then applied to investigate the aerodynamic contributions of the individual wing and body configurations using steady Reynolds-averaged Navier-Stokes (RANS) simulations. Finally, the full BBWT configuration is analysed over a broad range of airspeeds and inflow conditions representative of dynamic soaring to determine the aerodynamic response and identify the crosswind and downdraft limits beyond which sustained soaring is no longer possible. The findings are then discussed, and the main conclusions are presented.

## RESULTS

### Grey-headed albatross body component evaluation

**Fig. 1.**
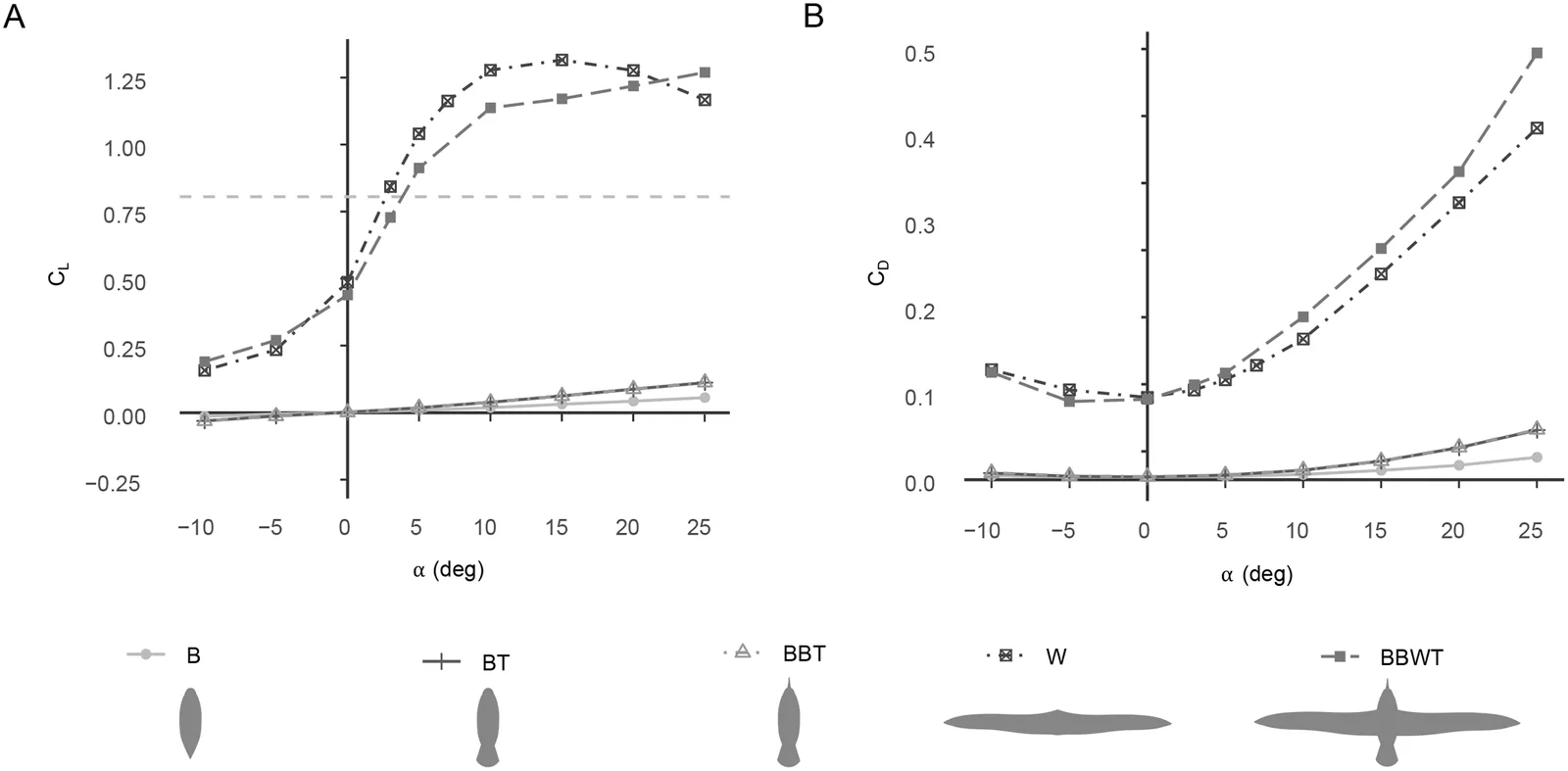
**Lift (C_L_; A) and drag coefficients (C_D_; B) versus angle of attack (α) at a freestream speed (U) of 15 m s^−1^ (Reynolds number of ca. 150,000) and sideslip angle (β) of 0° for all five configurations of the grey-headed albatross geometry.** The dashed line, at ca. C_L_=0.79, illustrates the condition where the lift generated equals the average mass of an adult grey-headed albatross, 3.3 kg (Schoombie et al., 2025b). Schematics of the body-alone (B), body-tail (BT), bill-body-tail (BBT), wing-alone (W) and bill-body-wing-tail (BBWT) configurations of the simulated geometry are included at the bottom.

The maximum contribution to lift production from the B, BT and BBT configurations represented only 7.1%, 14.2% and 14.3% of the assumed weight of the grey-headed albatrosses, respectively (Fig. 1). The addition of the tail significantly altered the lift slope, while the bill had negligible effect due to its small frontal area and streamlined shape in the vertical plane. As expected, the wing dominated lift production, generating lift that exceeds weight for α>3° (Fig. 1A). However, incorporating the body components, and thus removing a portion of the wing at the root to form the BBWT configuration, reduced lift at α>0°, and had the effect of delaying stall.

The body alone had a small C_D_ component (Fig. 1B), which is unsurprising as it resembles low drag bodies from (Smith et al., 2017). The addition of the tail increased the C_D_, particularly at non-zero α, though the bill had virtually no notable effect on C_D_ across all α. As expected, the wing also dominated the drag production, with minimum C_D_ at α≈0°, similar to a wing with a symmetric airfoil. When combining all components into the BBWT configuration, the minimum C_D_ was offset to a negative α (ca. −5°). Though the wings clearly dominate the lift production, the presence of the bill, body and tail have a notable effect on the load trends, justifying their inclusion in the model for the overarching aerodynamic investigation.

### Grey-headed albatross aerodynamic evaluation

**Fig. 2.**
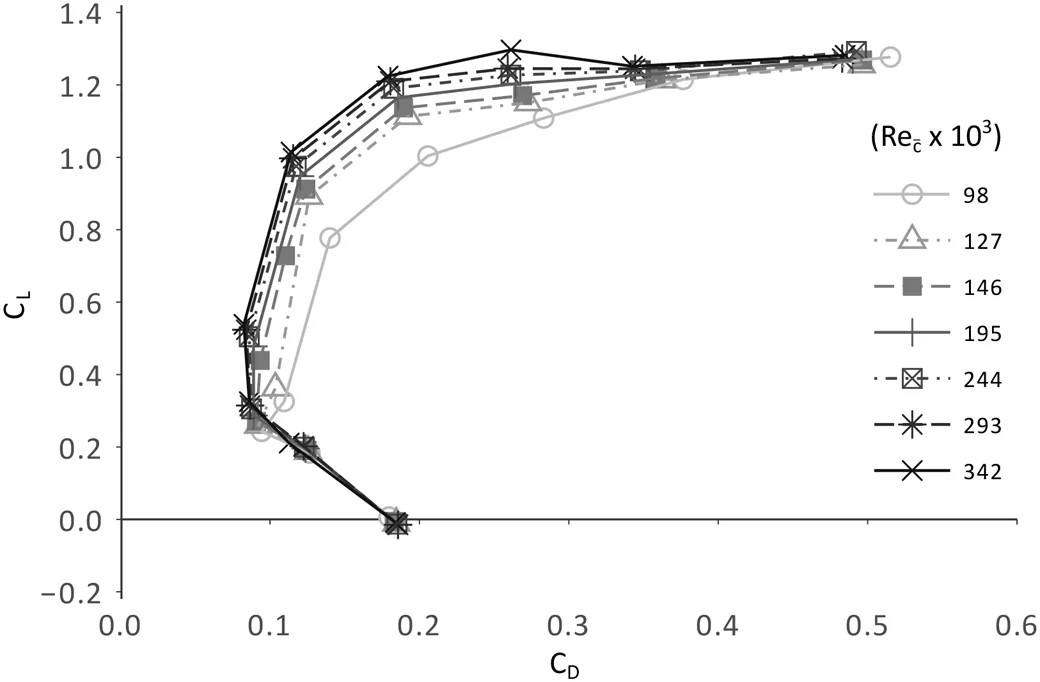
**Lift coefficient (C_L_) versus drag coefficient (C_D_) for varying angles of attack and chordwise Reynolds number,** 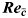
**, at a 0° sideslip angle.**

The C_L_ and C_D_ values for a given side-slip angle (β) showed a small, yet noticeable, Reynolds number dependence (Fig. 2). However, these differences decreased with increasing 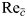 consistent with experimental observations for two-dimensional airfoils in a similar 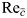 range (see e.g. Lyon et al., 1997). Apart from C_D_ at high α, both coefficients showed a marked dependence on β (to be discussed later), more so than 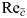. These results demonstrate that the present CFD model can be used to draw meaningful conclusions about the aerodynamics of the grey-headed albatross geometry.

**Fig. 3.**
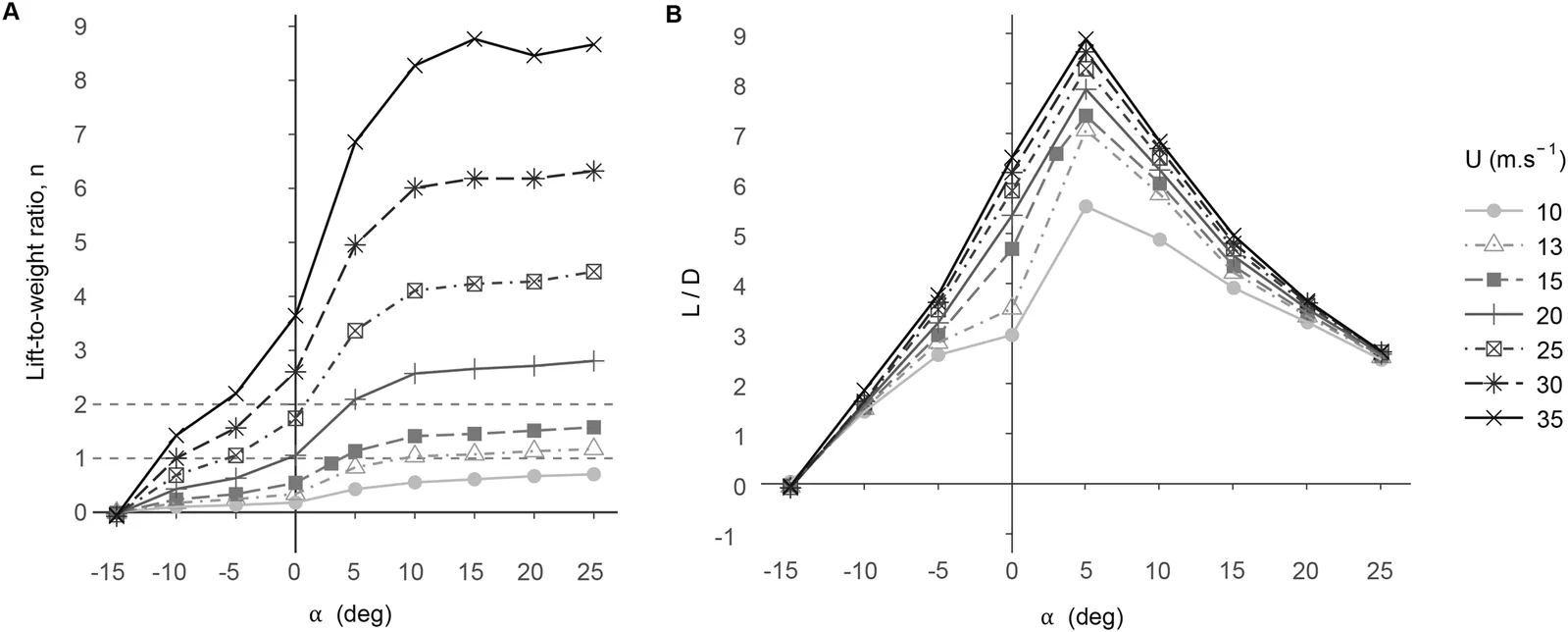
**Lift-to-weight ratio (n; A) and aerodynamic efficiency (L/D; B) plotted against angle of attack (α) for varying speeds (U) and constant sideslip angle (β) of 0°.** The dashed horizontal lines (A) indicate where sufficient lift is generated (n=1) and where lift exceeds the maximum load that the dried grey-headed albatross wing can withstand before non-linear deformation (n=2) according to estimations by Schoombie et al. (2025b).

**Fig. 4.**
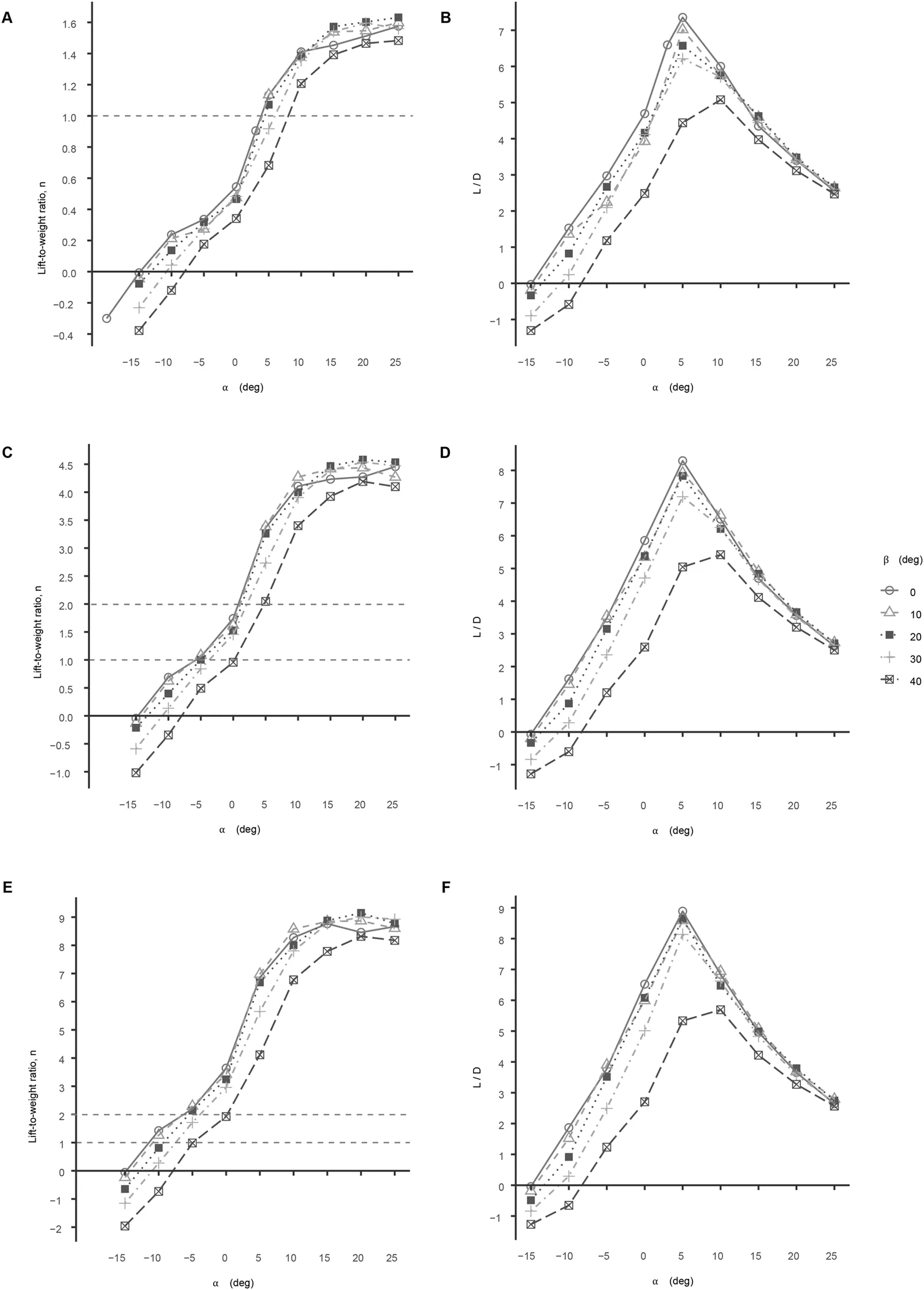
**Lift-to-weight ratio (n) and aerodynamic efficiency (L/D) plotted against angle of attack (α) for the grey-headed albatross model, bill-body-wing-tail (BBWT) configuration.** Sideslip angles (β) vary between 0° and 40°, at speeds (U) of 15 m s^−1^ (A,B), 25 m s^−1^ (C,D) and 35 m s^−1^ (E,F). The dashed horizontal lines (A,C,E) indicate where sufficient lift is generated (n=1) and where lift exceeds the maximum load that the dried grey-headed albatross wing can withstand before non-linear deformation (n=2; see Schoombie et al., 2025b).

The primary objective was to identify the combinations of U, α and β at which the albatross geometry generated insufficient lift to sustain flight. To achieve this, the lift-to-weight ratio (n) was plotted against α, highlighting regions where lift equalled weight (*n*=1), indicating adequate lift (Fig. 3). Additional simulations were run at α=−15° for β=0° and all U where lift became negative (Fig. 3A), indicating that, at α≤−15°, the grey-headed albatross geometry would experience a downward force across all β for speeds up to 35 m s^−1^. This albatross geometry generated lift up to nine times the weight of an adult grey-headed albatross, at U=35 m s^−1^ and α>10° (Fig. 3A). However, at U=10 m s^−1^, insufficient lift was generated even at high α. For U=13 m s^−1^ and 15 m s^−1^, lift exceeded weight for α>9°and α>3°, respectively, for β=0°. The aerodynamic efficiency (L/D) was plotted across α for all conditions to show where L/D is maximised and to assess the effect of U and β on the efficiency (Fig. 3B). Maximum L/D was achieved at α=5° for all U and β≤30° (Figs 3 and 4).

A grey-headed albatross in steady gliding flight will most likely need an airspeed between 20 and 25 m s^−1^, assuming a purely axial component of flow (α=0, β=0), to generate sufficient lift to counteract their weight when their wings are in the fully extended gliding position. In the absence of a crosswind (β=0°), with an updraft that maximises L/D (i.e. α=5°), the speed where L=F_w_ was interpolated to be ca. 14 m s^−1^, which is very similar to the average airspeed of 14.3 m s^−1^ estimated from field measurements (Schoombie, 2024) and falls within the airspeed range of 8.9–19.2 m s^−1^ as observed by Spear and Ainley (1997).

With no crosswind (β=0°), the albatrosses can most likely sustain flight even with a mild downdraft (α≥−5°), granted that the speed is high enough (U>25 m s^−1^), although the wing never appears to fully stall at these conditions (Fig. 3A). Sustaining flight at lower airspeeds is possible if there is at least a mild updraft (α>0°). However, if the updraft becomes too strong (e.g. α>5° for U>20 m s^−1^) the birds will need to retract their wings to reduce lift, as too high a load can cause injury. The threshold for this is most likely at L=2F_w_, as Schoombie et al. (2025b) estimated that the dried grey-headed albatross wing experienced non-linear deformation if a load of more than twice the weight is applied to the wing. In high winds grey-headed albatrosses were observed to retract their wings, reducing the wing area, particularly when flying into a headwind (personal observation, but see also e.g. Pennycuick, 1968). When there is a low wind (U<10 m s^−1^) or not enough of an updraft (e.g. α<5° for U=13 m s^−1^), then a power input will be required from the albatross, either through flapping, dynamic soaring or diving to generate sufficient lift.

**Fig. 5.**
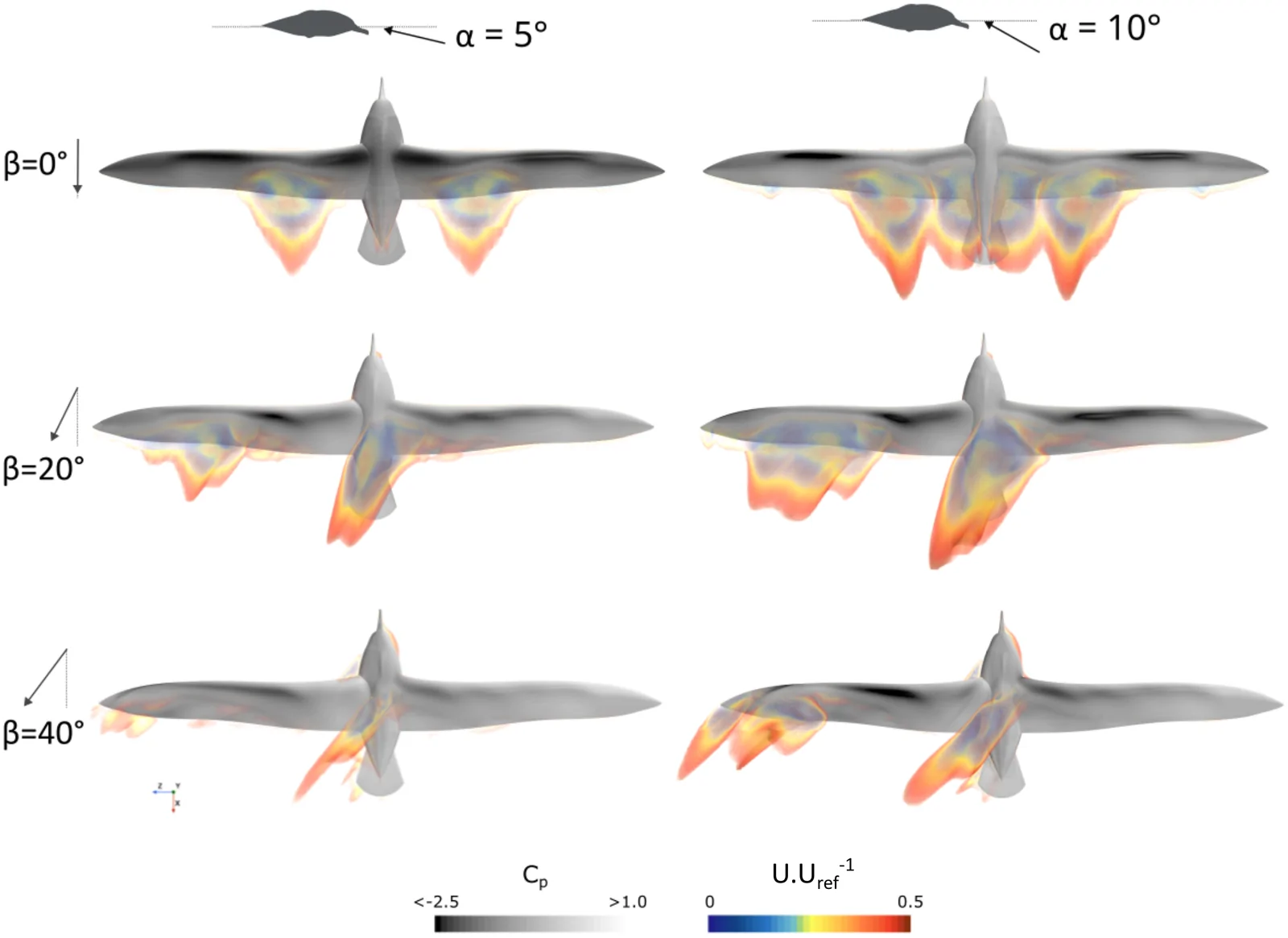
**Top view of bill-body-wing-tail configuration of the grey-headed albatross CFD model.** Colour contours show volumes of low relative velocity – local velocity magnitude (U) as a ratio of reference velocity (U_ref;_ i.e. freestream velocity magnitude at the CFD domain inlet) – which indicates separation. Surface contours indicate the calculated pressure coefficient (C_p_) on the grey-headed albatross model surface. Visualisations shown are for simulation conditions of U_ref_ of 15 m s^−1^ and the range of angles of attack (α) and side-slip angles (β) as indicated (not to scale).

At α<5°, an increase in β decreased lift for a given U (Fig. 4). At α>10°, the effect of increased β on lift can be positive or negative, likely due to the nature of flow separation at these higher sideslip angles – there appears to be less separation at β=40° compared to β=20° for certain positive α (Fig. 5). For U=15 m s^−1^, there was only a marginal decrease in lift at β≤20° at α≤10° (Fig. 4A), but a marked increase in lift for α>10°. At β=40°, lift can fall below the weight at a lower α compared to lower β, and produces negative lift at α<−8°. At U=25 m s^−1^ insufficient lift is generated at α<−5° with sideslip angle up to 30° (Fig. 4C) and n>2 for α≥0°. With a higher sideslip, β=40°, a strong negative lift (n≈−1) is generated at α=−15°, with positive lift (n≥1) only achieved at positive angles of attack (α>0°). At this speed, the geometry could generate lift up to n>4 even at β=40°. At U=35 m s^−1^, the negative lift generated at α=−15° is even stronger, up to n=−2, compared to U=25 m s^−1^ (Fig. 4E). At this speed, sufficient lift was generated even at negative α and 0°<β<30°, and n>2 at α≥−5°.

For all speeds, L/D decreased with increasing β, with the maximum L/D at α=5° for β≤30°. At β=40°, the maximum L/D occurred at α≈10° for all speeds (Fig. 4B,D,F). The L/D – α relationship has a sharper curve than is typical for engineered aircraft (see e.g. Anderson, 2008). The maximum L/D is observed at α=5° for all speeds and sideslip angles except for β=40°. At this higher sideslip angle, there is more separation (and thus higher pressure drag) at 10°, but the pressure coefficient (C_p_) is lower resulting in the higher lift production compared to alpha=5° (Fig. 5, see also Fig. S6). At β=0°, the most separation occurs around the elbow joint, between 0.2≤Y≤0.4 m, where the cross-section is thicker and more cambered. As the sideslip angle increases, the separation zones shift downstream towards the wing tips and in the lee of the body (Fig. 5).

#### Effect of crosswinds

**Fig. 6.**
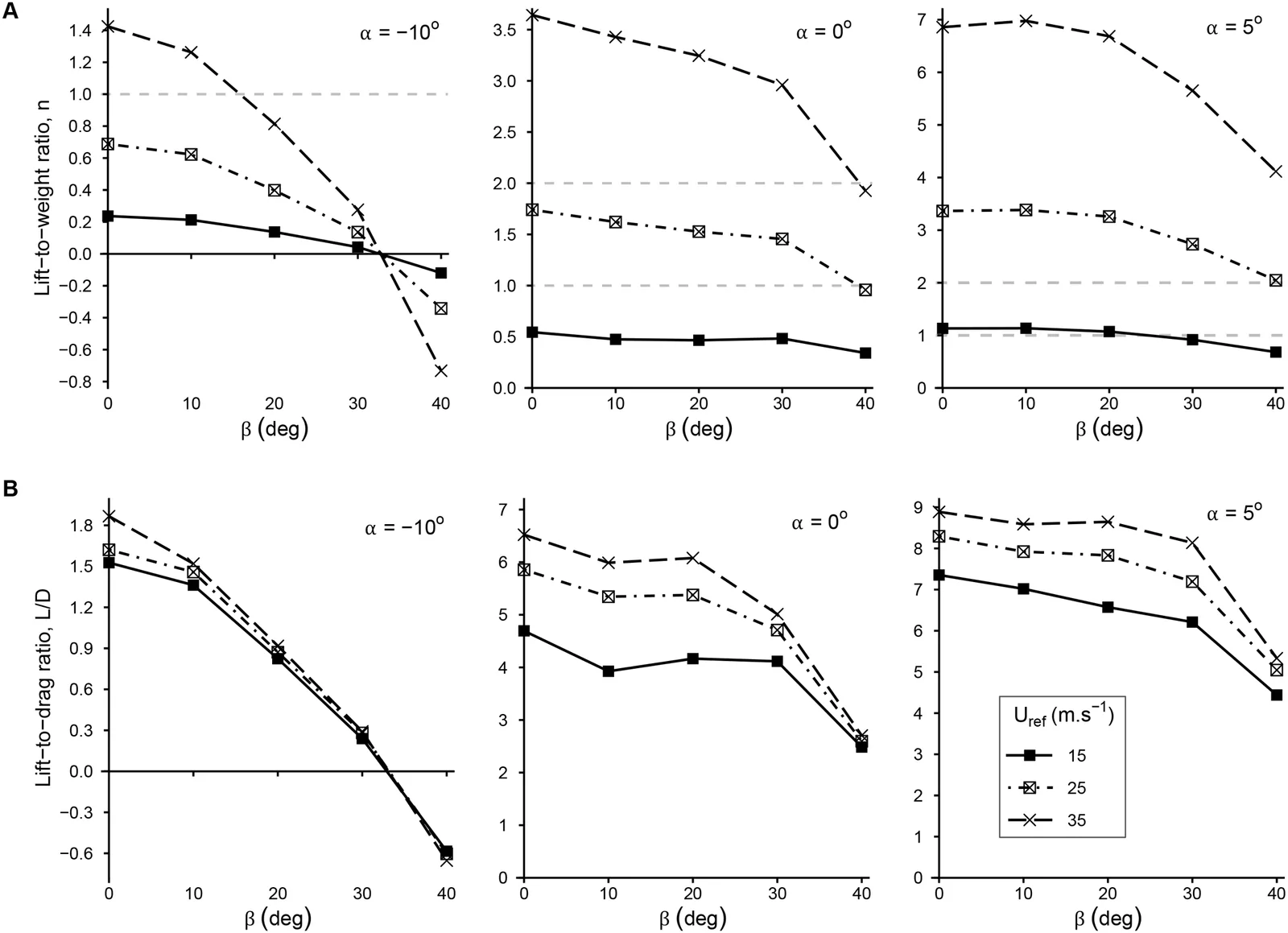
**The effect of sideslip angle (β) on lift-to-weight ratio (n; A) and lift-to-drag ratio (L/D; B), also known as the aerodynamic efficiency, for angles of attack (α) of −10°, 0°, and 5°, as indicated.** The dashed horizontal lines indicate where sufficient lift is generated (n=1) and where lift exceeds the maximum load that the dried grey-headed albatross wing can withstand before non-linear deformation (n=2; see Schoombie et al., 2025b).

Overall, n decreased with increasing β (Fig. 6A). At zero and negative angles of attack (α≤0°), a steeply decreasing trend is observed, and there is only enough lift generated (n≥1) at high airspeeds (U≥25 m s^−1^; Fig. 6A). At α=0° and U≥30 m s^−1^ the grey-headed albatross geometry generated more lift than twice the weight (i.e. n>2*)*, which exceeded the maximum load for the dried wing. It is thus possible that, for U≥30 m s^−1^ and β<30°, a grey-headed albatross in flight might need to retract its wings to reduce lift. At α=5° (Fig. 6A), lift equalled or exceeded weight (n≥1) at all airspeeds simulated (≥15 m s^−1^) at low sideslip angles (β≤20°), and exceeded 2F_w_ at U≥19 m s^−1^.

At negative angles of attack, the aerodynamic efficiency (L/D) is low and decreases sharply with increasing sideslip angle (β) (Fig. 6B). The glide ratio and sink rate, often used to quantify avian aerodynamic performance in seabirds (Pennycuick, 1982; Rosén and Hedenström, 2001; Van Bokhorst et al., 2015), are linked to L/D; a reduction in L/D leads to a higher sink rate and reduced glide range. At 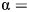
0°, L/D remains relatively stable for 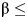
30° before decreasing at 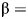
40° (Fig. 6B). At the angle of attack corresponding to maximum efficiency 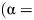
5°), the decline in L/D with increasing β is more gradual, although a pronounced drop is again observed at 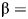
40°.

The majority of viable flight conditions occur near zero sideslip (β=0°), or zero crosswind component, while increasing β generally requires a higher angle of attack (α) or airspeed to maintain sufficient lift. E.g. if a grey-headed albatross were travelling at a groundspeed of 5 m s^−1^ and α=0° (zero up- or downdraft) and it experiences a headwind of ca. 24 m s^−1^, it's airspeed at that moment would be 29 m s^−1^, generating lift at double its weight (n>2; Fig. 4E) if its wings were fully extended. Similarly, a departing bird with a tailwind of 24 m s^−1^ would need a groundspeed of at least 44 m s^−1^ (ca. 158 km h^−1^) to get enough airspeed (20 m s^−1^ at α=0°) for sustained flight.

## DISCUSSION

### Aerodynamic causes of crash-landings

As is the case with all albatrosses, grey-headed albatrosses have evolved to specialise in soaring flight (Pennycuick, 1982; Weimerskirch et al., 2000; Richardson, 2011). Having long narrow wings and a high wing loading (Warham, 1977; Suryan et al., 2008) would imply that albatrosses require at least low winds to be able to take off (Uesaka et al., 2023). Their wing morphology also makes manoeuvres such as landing and taking off more difficult (Weimerskirch et al., 2000; Videler, 2005) compared to other birds. Grey-headed albatrosses breed on cliffs, which enables them to dive and gain sufficient airspeed immediately after take-off. At an inland breeding colony on Marion Island, an east-facing ridge, grey-headed albatrosses frequently encounter tailwinds during take-off, resulting in prolonged low-altitude flight over rugged terrain before reaching the ocean (Schoombie et al., 2023a,b). The predominantly westerly winds further generate recirculating flow in the lee of the ridge, producing highly variable wind vectors that may be linked to adult mortality. The present study quantified the combinations of airspeed, angle of attack, and sideslip angle induced by such winds that may lead to fatal crash-landings.

At low airspeeds, the lift simulated for the CFD model did not generate sufficient lift to counteract the average weight of an average adult grey-headed albatross at any of the simulated angles of attack. The minimum speed where lift equals weight (n=1), for the optimal L/D (at α=5° and β=0°), was ca. 14 m s^−1^. This falls well within the expected range of airspeed estimated for seabirds in general (typically 10–18 m s^−1^; Nourani et al., 2023). The model can also generate lift up to nine times the weight of an average adult grey-headed albatross at high airspeeds and strong updrafts.

At even moderate sideslip angles and relatively low speeds, the albatross model generated sufficient lift only at positive α (e.g. at U=15 m s^−1^ and α>5°). Thus, with a moderate crosswind and a low airspeed, a gliding grey-headed albatross would likely require a moderate updraft to generate enough lift to maintain gliding flight. Thus, in a crosswind or turning manoeuvre with no updraft, the gliding bird would need to increase its airspeed (either by turning into the wind or diving), increase its angle of attack (by pitching up or tilting its wings), or flap to ensure it generates enough lift to stay airborne. With a strong crosswind and low airspeed, the lift on the grey-headed albatross model becomes negative when there is a downdraft component. In a gliding grey-headed albatross, this would result in downward acceleration which would propel the bird towards the ground. In such a scenario, especially if such wind appears suddenly and the bird is close enough to the ground, flapping may not be sufficient to counteract this downward acceleration (see also Sato et al., 2009). This would most likely result in a hard impact landing, with the bird sustaining severe injuries. Evidence was found of injuries that would cause instantaneous death on impact (Schoombie et al., 2023a,b), such as a broken neck. If the bird sustained an injury such as a broken leg or wing and survived, it would most likely be taken by predators or in time die of its injuries.

On Marion Island, under the predominantly westerly wind regime surrounding the Grey-headed Albatross Ridge colony, flow recirculation is expected within the adjacent valley, with updrafts forming near the cliff faces and downdrafts occurring further downstream (Schoombie et al., 2023a,b). Birds may intermittently exploit updrafts to assist with take-off and landing, even under crosswind conditions. However, given the transient and turbulent nature of the flow in the lee of a bluff body such as the ridge, these updrafts are likely unpredictable. This variability may also explain observations of increased flight activity during easterly wind conditions, when persistent updrafts are present along the ridge. In contrast, strong westerly and north-westerly winds increase the likelihood of birds encountering downdrafts in combination with crosswinds, conditions that can generate substantial downforce. During such latency periods, birds retain some capacity for aerodynamic adjustment; however, observational evidence reported by Schoombie et al. (2023a,b) indicates that many individuals fly at very low altitude within the valley under these conditions. As a result, limited height above ground likely restricts the time available for corrective manoeuvres, a constraint exacerbated by the birds' large body size and long wings, which reduce manoeuvrability.

Previous studies indicate that at the meso-scale (kilometres and hours), albatrosses travelling long distances over the ocean preferentially exploit crosswinds (Weimerskirch et al., 2000; Richardson et al., 2018). In the meso-scale context, their heading is approximately perpendicular to the general wind direction, as this allows them to effectively use dynamic soaring. It was also shown that within a dynamic soaring cycle, at a much smaller scale (metres and seconds), wandering albatrosses spend the majority of their flight time in a bank (Schoombie et al., 2019; Schoombie et al., 2023a,b). However, at these finer scales, the aerodynamic analysis of the grey-headed albatross model suggests that crosswinds reduce effective airflow over the wings, thereby decreasing lift generation and aerodynamic efficiency. This suggests that the wings may inherently have a slightly positive α relative to their line of travel, so that β can be quite large and they can still have enough lift and a long gliding range (low glide angle and sink rate).

An increase in the northerly component of the dominant wind direction and an increase in wind speed on Marion Island have been documented with long-term measurements from the meteorological station (le Roux, 2008). These changes are attributed to anthropogenic climate change and are observed throughout the Southern Ocean (Weimerskirch et al., 2012). While we know grey-headed albatrosses can ‘ride storms’ in the Southern Ocean (Catry et al., 2004), they face greater risks flying over land, as demonstrated in this study and in Schoombie et al. (2023a,b). The advantage of increased frequency of strong northerly winds could be that, with less frequent winds directly from the west, take-offs might be easier for a larger portion of the breeding season. This would reduce the likelihood of a bird losing too much altitude after take-off and being forced to fly low over the valley, thus reducing the possibility of fatal crash-landings. However, if wind speeds increase to the point that returning adults are unable to access their inland nests more frequently or for extended periods, this may disrupt adult breeding behaviour and, consequently, affect population dynamics on Marion Island.

### Uncertainties in CFD modelling

Because the geometry is assumed rigid, the simulations do not capture static wing deflections, active wing or tail adjustments to compensate for changing atmospheric conditions experienced by birds in flight. Instead, a uniform inflow and fixed tail configuration were assumed throughout. Although these simplifications limit the realism of the model, they allow the isolated effects of airspeed components on lift (and downforce) to be investigated, which is hypothesised to be a primary mechanism underlying crash landings in grey-headed albatrosses.

Although the geometry was reconstructed from an actual grey-headed albatross wing, it was obtained from a dried specimen. Wind tunnel testing showed greater flow separation, including feather lifting and small oscillations of the entire wing, than would be expected for a live bird in flight along Grey-headed Albatross Ridge. These effects may also have influenced the quality of the reconstructed geometry used in the CFD model.

Consistent with the wind tunnel observations, the CFD simulations predicted substantial flow separation over portions of the wing that is not typically observed in soaring albatrosses (Pennycuick, 2015; Schoombie et al., 2025b). This behaviour is likely associated with the increased thickness and camber introduced by the dried (stiffer) wing during geometry reconstruction, as previously noted by Schoombie et al. (2025b). Consequently, the maximum predicted L/D of 9, is lower than the theoretical estimates for albatrosses under ideal flight conditions, e.g. 21 for a wandering albatross (Pennycuick, 2008). These theoretical estimates are themselves based on assumed values of C_D_ under ideal physical conditions (Pennycuick, 2008), whereas the present CFD model resolves the aerodynamic forces directly from the reconstructed geometry. While the rigid CFD model therefore likely underestimates aerodynamic efficiency, no direct measurements of lift or drag currently exist for live grey-headed albatrosses in gliding flight against which these predictions can be validated.

Despite these limitations, the aerodynamic loads and trends observed in the CFD model of the grey-headed albatross geometry are sufficiently aligned with engineering expectations, e.g. the lift and drag curves resemble typical curves for a fixed-wing aircraft and coefficients show a reduced Reynolds number dependence with increasing Reynolds number, as observed in other reliable sources (Rumsey and Spalart, 2009). The CFD model therefore provides a credible framework for investigating the aerodynamic response of a grey-headed albatross maintaining a fixed gliding posture and identifying the combinations of airspeed components for which sufficient lift can no longer be generated.

## Conclusions

An aerodynamic investigation was conducted using a 3D CAD model of a grey-headed albatross in steady gliding flight. To our knowledge, this is the first study to quantify the aerodynamic limits of steady gliding grey-headed albatrosses to combinations of airspeed, angle of attack, and sideslip angle using computational fluid dynamics.

Comparison of the different body-component configurations showed that, although the wings dominate lift and drag production, the body, tail, and bill altered L-α trends and should therefore be included in biologically realistic aerodynamic models. The validated CFD framework was subsequently used to investigate the aerodynamic performance of the complete bird geometry over a range of flight conditions. The lift produced by the full grey-headed albatross model (bill, body, wing, and tail) has shown that grey-headed albatrosses have a morphology that is efficient at generating lift, even in the presence of a downdraft or crosswind, but not necessarily both at the same time. There are two scenarios that would force a grey-headed albatross to crash-land; 1) loss of lift through reduced airspeed and 2) downforce (negative lift) caused by strong winds with a high crosswind and downdraft component. Under these conditions, the model predicts a loss of aerodynamic performance sufficient to explain the crash-landings observed at the Grey-headed Albatross Ridge colony on Marion Island.

Loss of lift does not necessarily mean a hard impact landing but would mean some input is required to maintain flight. Though at meso-scale grey-headed albatrosses tend to favour crosswinds, the model suggests that strong winds with a high crosswind and low to moderate downdraft component together may drive a gliding grey-headed albatross into the ground, forcing a fatal crash-landing. Such conditions are most commonly experienced by the birds at departure in the dominant westerly winds, linking local wind structure to collision risk and highlighting the potential for broader impacts should prevailing wind regimes change. More broadly, this study demonstrates the value of combining biologically realistic digital models with computational fluid dynamics to investigate aspects of animal flight that cannot readily be measured in the field. Although the present model represents a simplified steady gliding configuration, the framework provides a foundation for future investigations incorporating wing morphing, unsteady atmospheric flows, and other seabird species.

## MATERIALS AND METHODS

All CFD simulations in this study used Siemens Simcenter STAR-CCM+ (v 18.04.008). No experimental aerodynamic data were available for a gliding grey-headed albatross, but the model methodology was validated against a benchmark case (detailed below) at an appropriate Reynolds number to establish confidence in the albatross CFD model within the constraints of the available information.

### Benchmark study

To verify the selected CFD methods, a three-dimensional finite wing with a NACA 0012 airfoil (wing cross-section) was analysed. In this process, a volume of air surrounding the wing geometry is discretised into a mesh of smaller cells. The fluid flow properties, such as velocity, pressure and temperature, are then obtained by iteratively solving the RANS equations and associated turbulence model equations throughout the mesh.

Preliminary calculations showed the estimated chordwise Reynolds number (Re_c_) range for a gliding grey-headed albatross is between 0.5×10^5^ and 2.5×10^5^. The benchmark case in Smith (2017) was conducted at a Re_c_ of 10^5^, which is comparable to the estimated average conditions experienced by a grey-headed albatross in flight. For this study, simulations on the NACA wing were done using the same domain shape as in Smith (2017; but see also Tank et al., 2017), and a mesh convergence check was executed (Fig. S1; but see also Schoombie, 2024), i.e. finding where increasing the number of cells in the mesh no longer yields a more accurate result. An important part of the meshing operation is discretising the boundary layer along the wing surface, which was composed of 20 prismatically extruded cell layers at a distance of 1.66 mm (or 0.022c) away from the wing surface (Table S1).

Although the benchmark case lies within the laminar flow regime, atmospheric turbulence is expected to drive flow over the grey-headed albatross model into the turbulent regime. We therefore evaluate the influence of different turbulence models on the predicted lift (C_L_) and drag coefficients (C_D_). The fully turbulent k–ω SST model underpredicts both coefficients at low angles of attack (α≤2°), however, it captures transition-like boundary layer behaviour and requires 25% less computational time compared to the transition model, used by Smith (2017; see also Fig. S2). The simulations for the NACA 0012 rectangular wing established a repeatable mesh selection strategy that balances solution accuracy and computational cost. The mesh convergence was done at a single α, but was found to be applicable across a range of angles of attack (Fig. S2). While Re_c_=10^5^ lies at the lower end of the expected range for the grey-headed albatross geometry simulations, it represents a conservative validation case, as agreement with experimental data is expected to improve at higher Reynolds numbers or with increased freestream turbulence (e.g. Yu et al., 2024).

### Grey-headed albatross aerodynamic model

The aerodynamic analysis of the grey-headed albatross was conducted numerically, using a three-dimensional computer aided design (CAD) model developed by Schoombie et al. (2025b). The appropriate meshing procedure and physics models were identified through the benchmark case. To maintain consistency, the domain shape and size were the same for simulations of all five grey-headed albatross geometry configurations. The inlet boundary was placed 50 m (ca. 25 times the wingspan, b) upstream of the wing, and the outlet boundary was 100 m (ca. 50 b) downstream of the wing (Fig. S3).

As the wing is the most geometrically complex component, with the boundary layer height expected to vary spanwise due to changes in airfoil shape and chord length, a single mesh suitable for the full range of speeds (U), angles of attack (α) and sideslip angles (β) was sought. Mesh independence was therefore assessed separately for the body (B), body-tail (BT), bill-body-tail (BBT), and wing (W) configurations (Table S2). The final mesh for the bill-body-wing-tail (BBWT) configuration, comprised ca. 22.5 million cells, using the specifications from each of the separate components (Table S2) and the boundary layer mesh height settings as determined from the benchmark case (see Figs S4 and S5).

The lift and drag coefficients (C_L_ and C_D_) for all five configurations were compared to evaluate the contribution of each component to these loads. All the coefficients were normalised using the same reference area, i.e the wing-alone projected area (see Eqns 1 and 2):
(1)

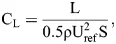

(2)

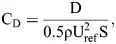
where L and D are the calculated lift and drag forces, respectively (in N), U_ref_ is the reference (or freestream) speed (in m s^−1^), ρ is the air density (in kg m^−3^), and S is the planform area of the wing (in m^2^). For the wing-alone configuration, the wing projected area (S) was determined to be 0.291 m^2^, with a wingspan (b) of 2.02 m (Schoombie et al., 2025b), resulting in a mean geometric chord 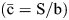 length of 0.1442 m. This value was used as the reference chord for the grey-headed albatross model unless stated otherwise.

The boundary and initial conditions for the albatross simulations were derived from measurements of actual environmental conditions taken at the relevant breeding colony and the meteorological station on Marion Island, located on the north-eastern coast of the island. These included a mean air temperature of 6.3°C, absolute pressure of 100,414 Pa, air density of 1.25 kg m^−3^, dynamic viscosity of 1.75×10^−5^ N s m^−2^, and turbulence intensity approximation of 35% (Table S3).

Simulations were conducted with boundary conditions of 10≤U≤35 m s^−1^ (which is the same as the reference velocity, U_ref_, unless stated otherwise) based on the minimum and maximum expected airspeeds, and −15°≤α≤25°. This range was based on the average up- and downdrafts measured on Marion Island (Table S3; but see also Schoombie, 2024), where adult grey-headed albatrosses were observed to crash-land, and to ensure the angle of attack is captured where C_L_ is zero and past the stall angle.

The word ‘root’ is used to refer to the section at the mid-plane (y=0 m) of the wing-alone geometry. Thus, the root angle of attack (α_root_) is the angle between the root chord line and the oncoming flow in the xz-plane. Since the twist angle between this wing-alone root section (y=0 m) and the section where the wing connects to the albatross body (y=0.01 m) is zero, the α_root_ will be the same for that section of the wing. This means that, when the body replaces the wing root for the BBWT configuration, there is no change in the reference α between configurations.

Since the albatross geometry is symmetric about the mid-body xz-plane, sideslip angles (β) ranged between 0° and 40° (all positive) with 10° increments. Non-zero sideslip angles were simulated for three representative speeds, U=15, 25 and 35 m s^−1^. This range was sufficient to provide information on the effect of crosswinds on lift generation and efficiency, which was the main aim of this numerical investigation.

Given the assumed high inherent turbulence in the air, 35%, the fully turbulent SST k-ω turbulence model was used in all albatross geometry configuration simulations. Solutions were considered converged when the residuals and calculated lift and drag coefficients (C_L_ and C_D_; see Eqns 1 and 2) converged to a constant value and no velocity or pressure gradients were observed at the domain boundaries (∼3500 iterations in all cases) and the mean of the last 200 iterations of C_L_ and C_D_ are reported as the result.

### Ethics

This research was done in compliance with all ethics regulations as stipulated by the University of Pretoria's Animal Ethics Committee and the Faculty of Engineering, Built Environment and IT.

### AI usage declaration

Large language models – ChatGPT (Open AI) and Le Chat (now Vibe, Mistral AI) – were used only to suggest language improvements in the writing of certain parts of the manuscript. All original ideas, opinions, and conclusions are those of the authors and were not generated by any AI tools. No AI tools were used to collect or analyse data, to write code, or to produce or edit any figures.

## Supplementary Material

10.1242/biolopen.062556_sup1 Supplementary information
