# Supplementary material for: Aerodynamic limits of gliding flight in grey-headed albatrosses under variable wind conditions
Source: Biol Open. 2026 Sep 10;15(9):bio062556. doi: 10.1242/bio.062556 (PMC13602366; doi:10.1242/bio.062556)
Supplement: Supplementary file 1 [file biolopen-15-062556-s1.pdf]

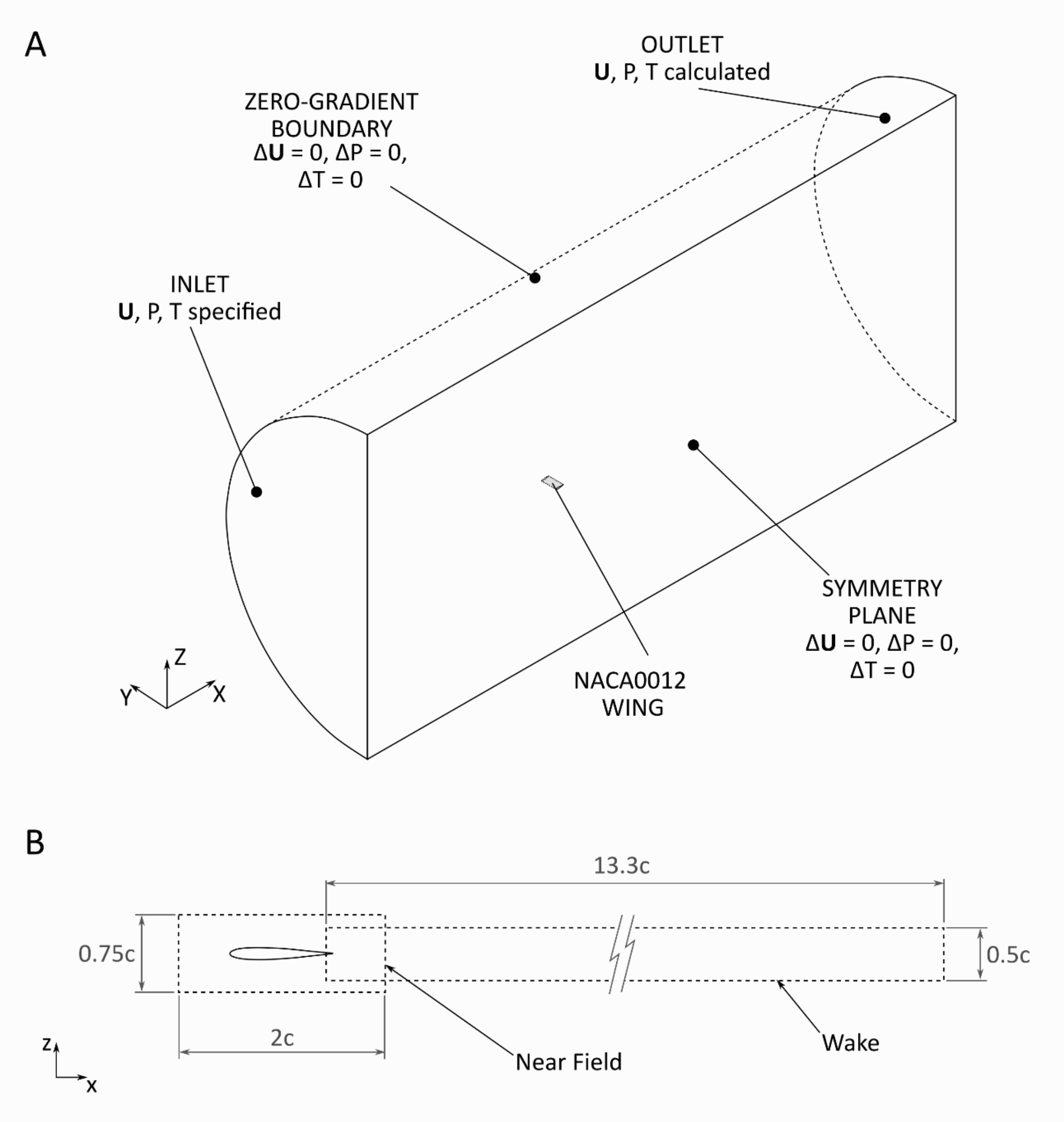

Fig. S1. **Graphical illustrations of the benchmark study model.** (A) The benchmark study geometry and domain shape. A finite rectangular wing with a NACA 0012 airfoil, chord length (c) of 0.075 m and span (b) of 0.28 m was simulated to validate the methods for simulating the grey-headed albatross geometry. The benchmark wing is rectangular and thus the chord length (c) the same along the span. A symmetrical cylindrical domain with a radius of 18c was used, mirroring that of Smith (2017). The inlet boundary was 25c upstream of the wing, and the outlet boundary was 50c downstream of the wing. (B) The fluid volume was discretised using polyhedral cells, featuring larger cell sizes at the far-field boundaries and refined zones near the wing (near-field) and in its wake. To test how the mesh refinement affects the solution, three meshes were created with cell counts of 1.7×10^6, 3.6×10^6 and 6.9×10^6, respectively. The mesh convergence test was completed for two turbulence models. For both closure schemes, the simulated results from the medium mesh (3.6×10^6 cells) were found to be within the asymptotic range, with a difference of < 1 % for the lift and drag coefficients compared to the fine mesh (6.9×10^6 cells). Thus, for the final mesh, the wing surface and trailing edge cell sizes were 0.5c and 0.25c respectively and the near field and wake regions were refined to 4.1c and 5c respectively.

Table S1. The wall y+ is a measure of how accurately the boundary layer mesh has captured the viscous sublayer of the boundary layer (White, 2008) and was used to determine the appropriate first cell height (y_h). In this study, we aimed for a y+ ≤ 1, explicitly solving the viscous sublayer (Siemens Digital Industries Software, 2023). For a constant y_h in the boundary layer mesh, there will be a variable friction velocity u_*, and thus variable wall y+ over the airfoil surface. To determine the optimal y_h, it was first estimated for y+ of 1, then incrementally decreased until the y+ across the whole surface was less than or equal to 1. The initial value of y_h is estimated based on the flat plate assumptions and an estimate of the friction velocity, $u_* = \sqrt{\tau_w/\rho}$ (in m.s^{-1}), the wall shear stress, $\tau_w = 0.5\rho U^2 C_f$ (in Pa) and the friction coefficient, $C_f = 0.664/\sqrt{Re_c}$ (assuming laminar boundary layer regime; White, 2008). Calculations in this table are given for a chord length (c) of 0.075 m and chordwise Reynolds number (Re_c) of 10^5.

Quantity	Value	Formula/Method
Chordwise Reynolds number, Re_c	10^5	$\frac{\rho U c}{\mu}$
Boundary layer height, δ	1.66×10^{-3} m (or 0.022c)	$\begin{cases} \frac{5c}{Re_c^{1/2}} & \text{laminar} \\ \frac{0.16c}{Re_c^{1/7}} & \text{turbulent} \end{cases}$
First cell height, y_h (Initial)	2.3×10^{-5} m ($y^+ = 1$)	$\frac{y^+ \mu}{\rho u_*}$
First cell height, y_h (Final)	1.0×10^{-5} m	Reduced iteratively until $y^+ \leq 1$ across entire wing surface

Biology Open • Supplementary information

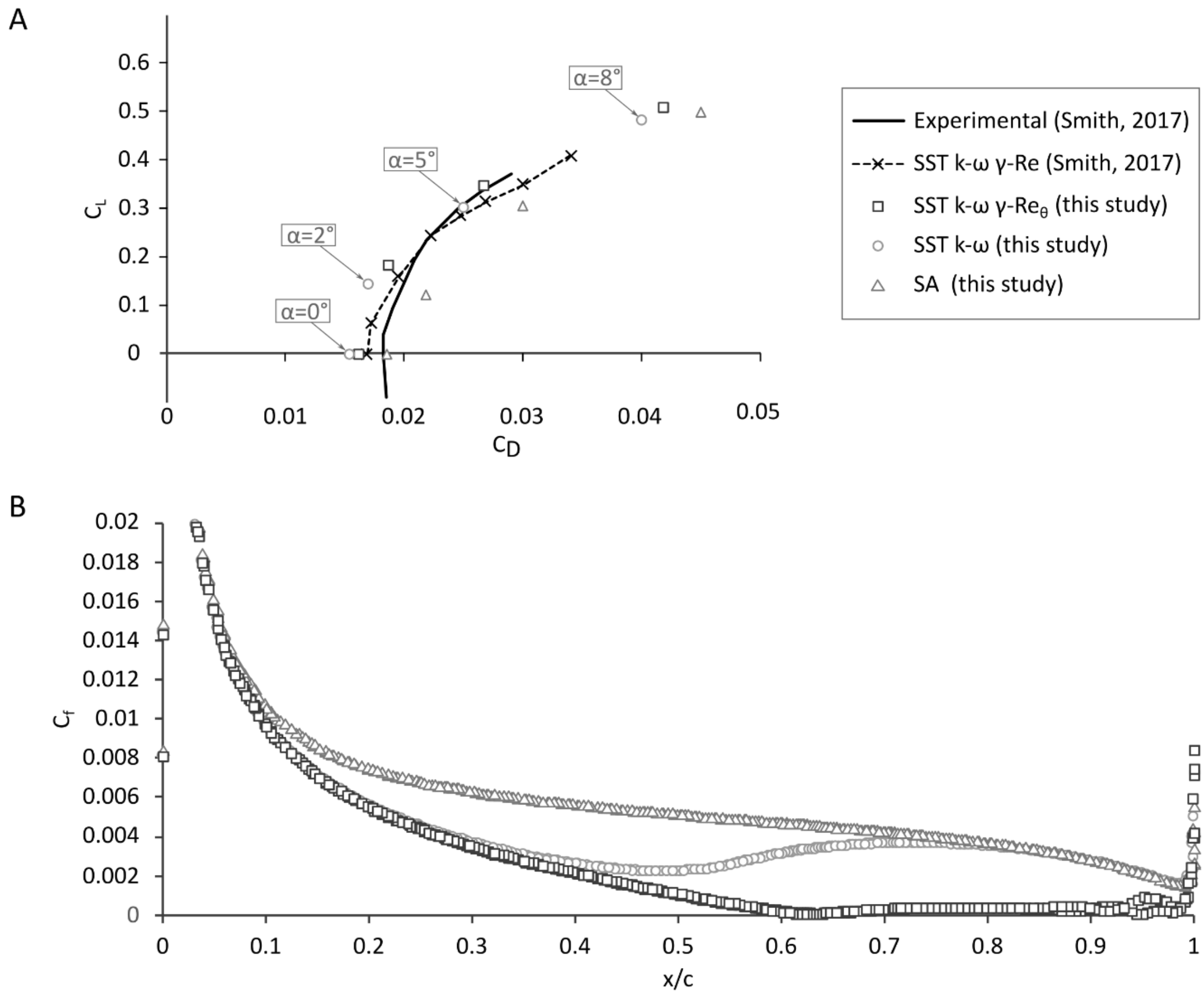

Fig. S2. Results for the benchmark study simulations. (A) Lift (C_L) versus drag coefficient (C_D) of the benchmark wing with NACA 0012 airfoil, for a range of angles of attack (α; indicated) at $Re_c = 10^5$. Results from this study are compared to experimental data and numerical simulations with three different turbulence models from Smith (2017): Spalart-Allmaras (SA), Shear Stress Transport k-ω (SST k-ω) and SST k-ω γ-Re_θ. B: Skin friction coefficient (C_f) plotted along the normalised chord (x/c) for the benchmark wing midline at α = 0° for the three turbulence models tested. The simulation results for both SST k-ω and SST k-ω γ-Re_θ models showed drag was under-predicted at low angles of attack, and over-predicted at higher angles of attack. The SA turbulence scheme over-predicted C_D at all angles of attack, though the C_L results did not differ markedly from the SST k-ω results. The SST k-ω model correlated well with the experimental data at $\alpha \geq 5°$, but errors increased at lower α. However, both the SST k-ω and k-ω γ-Re_θ results compare well to the SST k-ω γ-Re_θ RANS simulation data from Smith (2017). The largest difference in aerodynamic loads between the three simulated turbulence models occurs at α = 2°. The C_D predicted by the SST k-ω and S-A schemes, compared to the SST k-ω γ-Re_θ scheme differed by 9% and 17%, respectively. The C_L predicted by the SST k-ω and S-A schemes, compared to the SST k-ω γ-Re_θ scheme differed by 20% and 32%, respectively. At $\alpha \neq 2°$, the errors are < 6% for both C_L and C_D for the SST k-ω model. (B) Skin friction coefficient (C_f) along the x-direction over the upper surface of the wing, mid-span, for the three different turbulence models. Despite the subcritical Re_c, the SST k-ω model remains advantageous as it can estimate boundary layer transition, similar to behavior observed in Rumsey and Spalart (2009) and is more robust in separated flow simulations (Siemens Digital Industries Software, 2023).

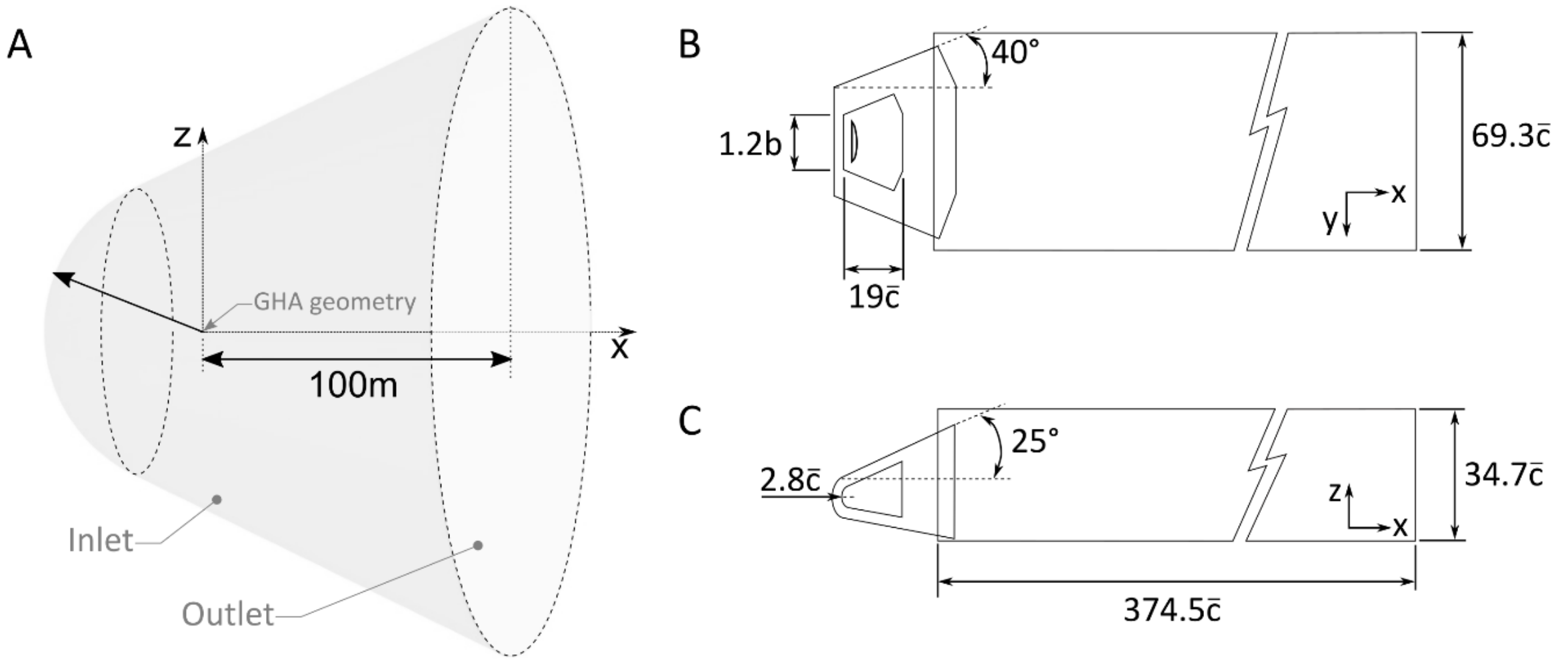

Fig S3. Graphic illustration of the grey-headed albatross model. (A) Frustum-shaped domain for the grey-headed albatross computational fluid dynamics (CFD) model. The CFD strategies validated by the benchmark case (boundary layer mesh setup, mesh convergence process, domain size, and physics solvers) were applied to the grey-headed albatross geometry to obtain a robust CFD model. Unlike the benchmark case, the albatross geometry was simulated at a much wider range of angles of attack and sideslip angles, and thus the domain shape was modified to avoid computational errors at the inlet boundary. As the domain boundaries were located sufficiently far from the model, the change in domain shape did not impact results. (B & C) Top and side view diagrams showing the dimensions of refinement regions. Dimensions are given either as a fraction of the grey-headed albatross wing mean geometric chord ($\bar{c}$) or wingspan (b). Mid-field and near-field mesh refinement volumes were designed to expand relative to the expected angle of attack and sideslip angle ranges to capture the pressure gradients near the wing surface, which were expected to be greater and extend further away than for the body components. The mid-field volume has the same shape as the near-field volume, scaled up by a factor of 2. The wake refinement volume extended up to 50 m downstream of the wing leading edge. These refinement regions were maintained for all configurations. Note that mean geometric chord ($\bar{c} = \text{wing area} \ / \ \text{wing span}$) is used as a reference as the chord length varies for the albatross wing, from largest at the root to zero at the tip.

A

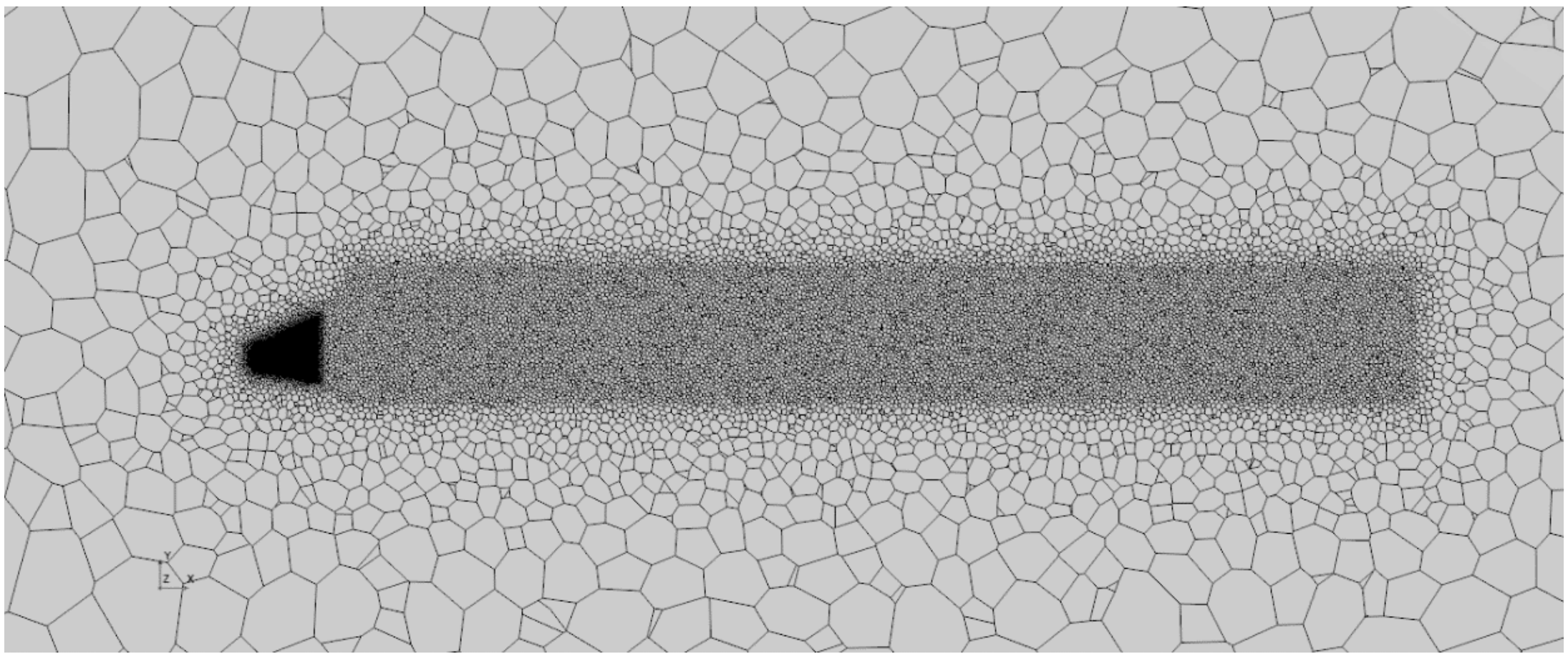

B

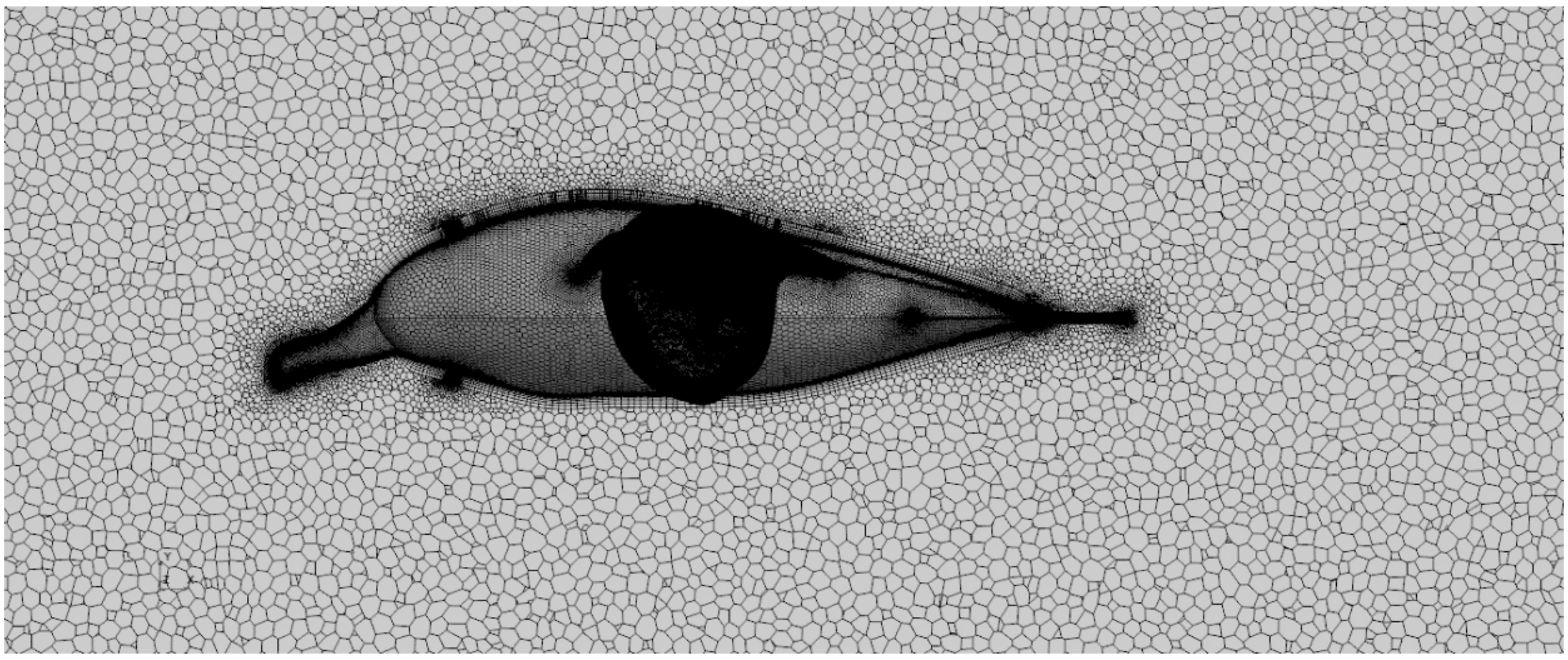

C

$$\delta_{\text{GHA wing}} = \max\left(\frac{5\bar{c}}{{\text{Re}_{\bar{c},min}}^{0.5}}, (\delta/c)_{\text{NACA0012}} \times \bar{c} \right)$$

Fig. S4. **(A) Mesh refinement regions for the grey-headed albatross (GHA) bill-body-wing-tail (BBWT) configuration and (B) an enlarged region showing surface refinement on the BBWT components**. For the benchmark case, the boundary layer height (δ) estimation from the laminar flat plate equation successfully captured the boundary layer, with a height of 0.022c. Since δ increases with a decrease in chordwise Reynolds number ($\text{Re}_{\bar{c}}$), and to ensure the boundary layer mesh captures velocity gradients, the maximum of two calculations (C) was used for the boundary layer mesh height for the grey-headed albatross wing mesh ($\delta_{\text{GHA wing}}$), where $\text{Re}_{\bar{c},min}$ is the Reynolds number relative to the wing geometric chord ($\bar{c}$), calculated at the minimum simulated airspeed (10 m·s^{-1}), and $(\delta/c)_{\text{NACA0012}}$ is the boundary layer height as a fraction of its chord (c) for the NACA 0012 benchmark case. The boundary layer mesh was also visually inspected to confirm that the velocity profile within the boundary layer was adequately captured.

Biology Open • Supplementary information

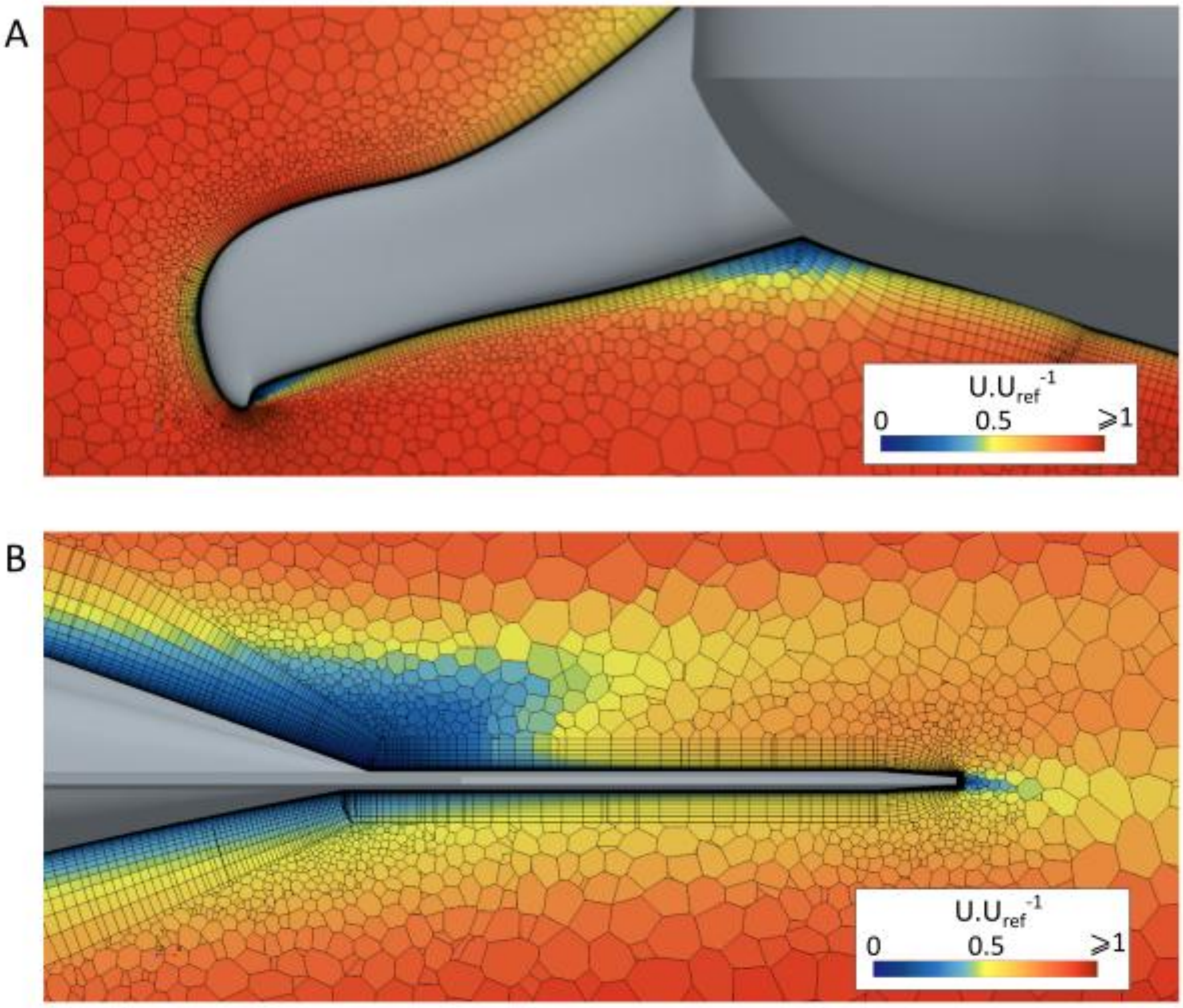

Fig. S5. Boundary layer mesh for the bill (A) and tail (B) components demonstrating the boundary layer mesh height necessary to capture flow features. Colour contours show the simulated speed (or velocity magnitude; U) at each mesh cell as a ratio of the reference (or freestream) speed (U_{ref}). Accurately resolving boundary layer behaviour is critical, making it a primary focus during meshing. The construction of the boundary layer mesh requires at least three inputs: the wall height (y_h; i.e., the height of the cell adjacent to the wing wall), the total boundary layer height (δ), and the number of cells within the layer. The boundary layer mesh was created using orthogonally extruded surface cells in 20 layers for the application of low y+ wall functions (i.e. $y+ \leq 1$). (A) The bill boundary layer mesh height was set to 3.5 mm, which is greater than the calculated δ = 1.9 mm for a bill length of 105.2 mm, to better capture the recirculating flow behind the bill tip. (B) The tail boundary layer mesh height was set to 3.5 mm, which is greater than the calculated δ = 2.4 mm for a tail length of 0.16 m to capture the velocity gradients caused by separated flow at the body-tail juncture.

Table S2. Mesh description for the grey-headed albatross bill-body-wing-tail (BBWT) configuration CFD model: boundary layer mesh height and cell sizes on the relevant surfaces and volume refinement zones. The surface and volume mesh for each of the body-alone (B), body-tail (BT), bill-body-tail (BBT) and wing alone (W) configurations were tested separately for mesh dependence. Mesh convergence was investigated using the halved domain (i.e. symmetric about the xz-plane; see Fig S3) and the SST k-ω turbulence model. Three meshes with increasing cell count were simulated at 5° angle of attack, 0° sideslip angle and freestream velocity of 15 $m \cdot s^{-1}$ for each configuration. The first wall-adjacent cell height (y_h) was 0.01625 mm on all surfaces resulting in a surface-averaged y+ of 0.3, indicating the viscous sublayer was adequately resolved. Differences in all aerodynamic loads were < 2% between the fine and medium meshes, and the results from these meshes were in the asymptotic range, indicating the medium mesh was adequate for the simulations. Inspecting the mesh and pressure gradients showed that the regions of high gradients were fully captured within the near-field mesh refinement zone. For the BBWT configuration, the surface, volume, and boundary layer mesh settings from the W and BBT medium meshes were combined into a single mesh (convergence test not repeated). All sizes/lengths are given in metres (m) or as a fraction of the mean geometric chord ($\bar{c}$).

Refinement zone/surface	Target cell size		Minimum cell size		Boundary layer mesh height
	m	Relative to $\bar{c}$	m	Relative to $\bar{c}$	m
Bill	2.45×10^{-3}	1.7%	2.9×10^{-4}	0.2%	3.5×10^{-3}
Body	4.9×10^{-3}	3.4%	2.9×10^{-4}	0.2%	12.0×10^{-3}
Wing surface	2.9×10^{-3}	2%	2.9×10^{-4}	0.2%	3.2×10^{-3}
Wing leading edge	2.9×10^{-4}	0.2%	2.9×10^{-4}	0.2%	3.2×10^{-3}
Tail surface	2.45×10^{-3}	1.7%	2.9×10^{-4}	0.2%	3.5×10^{-3}
Wing and tail trailing edge*	2.9×10^{-4}	0.2%	2.9×10^{-4}	0.2%	-
Near-field volume	0.012	8%	-		-
Mid-field volume	0.1442	100%	-		-
Far-field boundary	5.8	4000%	4.3	3000%	

*Wing is truncated at 99% chord. This refinement creates at least two cells on the trailing edge surface

Table S3. Summary of atmospheric conditions for Marion Island, measured from the meteorological station between January 2017 and December 2020 (data obtained directly from the South African Weather Service), which is ca. 15 km north-east of the study site (see also Goddard et al., 2022; Schoombie, 2024, and Schoombie et al., 2025). Wind speed and direction are measured at 10 m above ground. Density was calculated using ideal gas law and dynamic viscosity was calculated using Sutherland's law, with constants R = 287 $J.kg^{-1}.K^{-1}$, μ_0 = 1.716 x 10^{-5} $N.s.m^{-2}$ and T_0 = 273.11 K (White, 2006). The turbulence intensity (TI) is calculated based on the three-dimensional wind velocity magnitude, $|U_w|$, and the standard deviation of the wind velocity x-, y- and z-components of the standard deviation (σ_x, σ_y, and σ_z respectively). The three-dimensional wind vectors were measured at the study site, near Grey-headed Albatross Ridge (see Rudolph et al., 2022), using a RM Young 3D Ultrasonic Anemometer at a height of 2m above ground. Wind vectors were recorded at 4Hz for the purpose of estimating the TI near the albatross nesting site and flight paths. Wind direction and the vertical component could also be calculated for the study site using trigonometric relations between the vector components. At this location and height above ground (i.e. 2 m.a.g.l.), the strongest downdrafts were most often associated with south-westerly winds (ca. 200°) and secondly with north-westerly winds (~300°), both of which are the dominant direction modes at this location (Schoombie et al., 2023). The strongest updrafts are also largely associated with more easterly winds.

Property	Units	Value (± SD)	Formula
Dry bulb temp (T)	°C (K)	6.3 ± 3.0	--
Pressure (P)	Pa	100 414 ± 11.5	--
Wind speed (U_w)	$m.s^{-1}$	8.3 ± 4.5	--
Mean gust speed	$m.s^{-1}$	12.2 ± 6.2	--
Maximum gust speed	$m.s^{-1}$	59.6	--
Wind direction	°	291.6 ± 1.1	--
Density (ρ)	$kg.m^{-3}$	1.25	$\frac{P}{R\,T}$
Dynamic viscosity (μ)	$N.s.m^{-2}$	1.75 x 10^{-5}	$\mu_0\left(\frac{T}{T_0}\right)^{\frac{3}{2}}\frac{(T_0+111)}{(T+111)}$
Turbulence intensity (TI)	--	0.35	$\frac{\sqrt{\sigma_x^2+\sigma_y^2+\sigma_z^2}}{\sqrt{3}\,\lvert U_w\rvert}$

Biology Open • Supplementary information

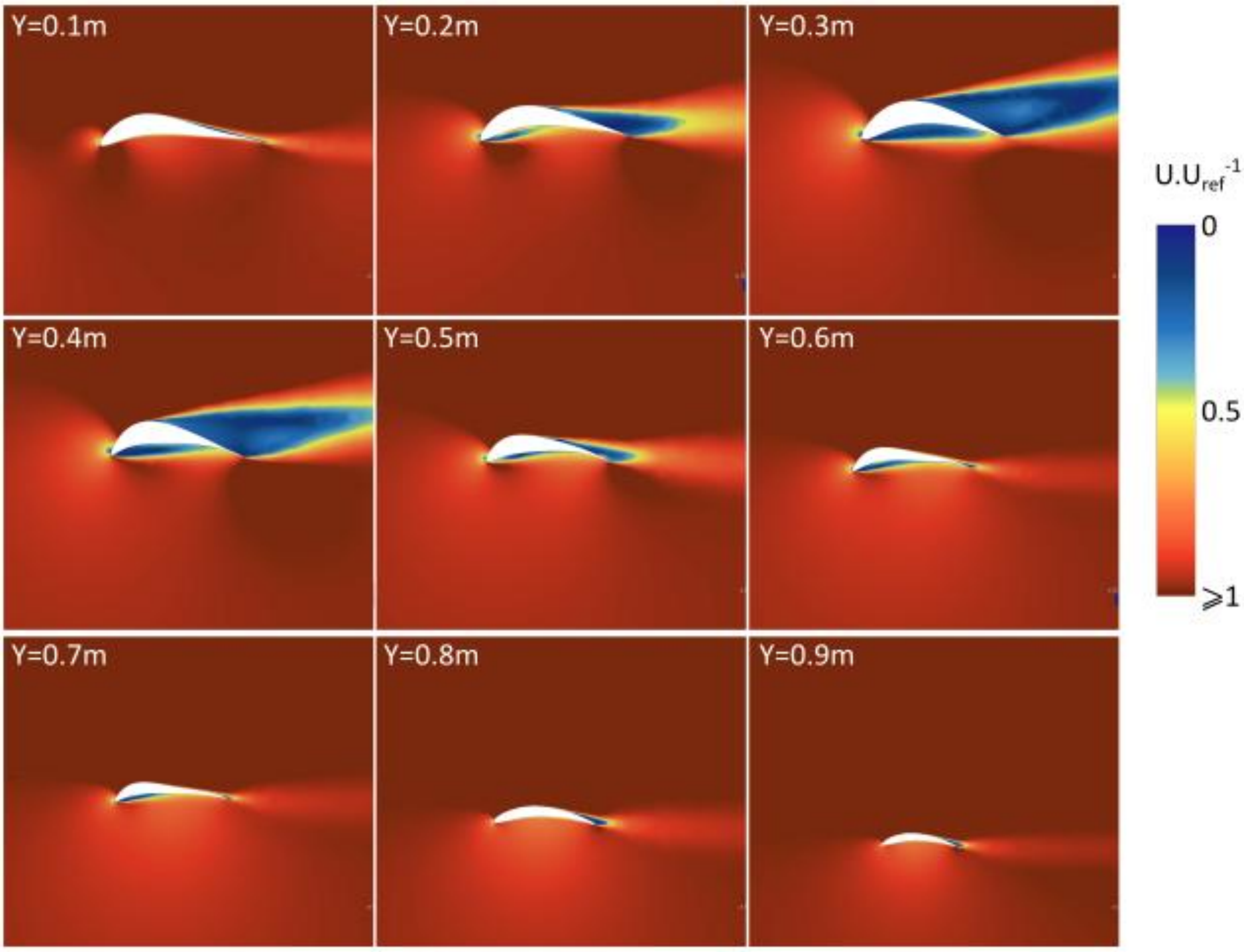

Fig. S6. Smoothed colour contours velocity magnitude (U) as a ratio of the reference velocity (U_{ref}) at y-normal plane sections at 0.1 m intervals (as indicated) along the grey-headed albatross wingspan. These are the simulated velocity fields for a U_{ref} = 15 m.s^{-1}, 5° angle of attack and zero sideslip. The low velocity wake between 0.2 m and 0.4 m indicate separated flow around the wing elbow joint, which increases pressure drag. Despite the presence of this separation, which is not observed in nature, this condition (at 5° angle of attack) produces the maximum aerodynamic efficiency (lift-to-drag ratio) for the grey-headed albatross geometry.

Additional References

Goddard, K.A. et al. (2022) "Investigation of ecologically relevant wind patterns on Marion Island using Computational Fluid Dynamics and measured data," Ecological Modelling, 464, p. 109827. Available at: https://doi.org/https://doi.org/10.1016/j.ecolmodel.2021.109827.

Rudolph E. M., Hedding D. W., de Bruyn P. J. N., & Nel W. (2022) "An open access geospatial database for the sub-Antarctic Prince Edward Islands," South African Journal of Science, 118(9/10), p. 12302. Available at: https://doi.org/10.17159/sajs.2022/12302.

Biology Open • Supplementary information

Rumsey, C.L. and Spalart, P.R. (2009) “Turbulence model behavior in low Reynolds number regions of aerodynamic flowfields,” AIAA Journal, 47(4), pp. 982–993. Available at: https://doi.org/10.2514/1.39947.

Schoombie, J., Schoombie, S., Connan, M., Jones, C. W., Risi, M., Craig, K. J., Smith, L., Ryan, P. G., & Shepard, E. L. C. (2023) “Impact of wind on crash-landing mortality in grey-headed albatrosses *Thalassarche chrysostoma* breeding on Marion Island,” Marine Ecology Progress Series, 723, pp. 213–225. Available at: https://doi.org/10.3354/meps14292.

Schoombie, J (2024). Investigating aerodynamic factors in wind-driven grey-headed albatross crash-landings. PhD Thesis, University of Pretoria, Pretoria, South Africa. Available at: http://hdl.handle.net/2263/99000

Schoombie, J., K. J. Craig, K. A. Goddard, D. W. Hedding, W. Nel, and P. C. le Roux. "Intra-Island Variation in Wind Patterns on Sub-Antarctic Marion Island." Geoscience Data Journal 12, no. 4 (2025): e70035.

Siemens Digital Industries Software (2023) Simcenter STAR-CCM+ User Guide.

White, F.M. (2006) Viscous Fluid Flow. 3rd ed. McGraw-Hill Higher Education (McGraw-Hill international edition).

White, F.M. (2008) Fluid Mechanics. 6th ed. New York: McGraw-Hill.

Biology Open • Supplementary information
